# Genome-Wide Identification and Expression Analysis of the *NRAMP* Family Genes in Tea Plant (*Camellia sinensis*)

**DOI:** 10.3390/plants10061055

**Published:** 2021-05-25

**Authors:** Jinqiu Li, Yu Duan, Zhaolan Han, Xiaowen Shang, Kexin Zhang, Zhongwei Zou, Yuanchun Ma, Fang Li, Wanping Fang, Xujun Zhu

**Affiliations:** 1 College of Horticulture, Nanjing Agricultural University, Nanjing 210095, China; 2018104081@njau.edu.cn (J.L.); 2018204034@njau.edu.cn (Y.D.); hanzl@njau.edu.cn (Z.H.); 2019104081@njau.edu.cn (X.S.); 2019104086@njau.edu.cn (K.Z.); myc@njau.edu.cn (Y.M.); lifang@njau.edu.cn (F.L.); fangwp@njau.edu.cn (W.F.); 2 Department of Plant Science, University of Manitoba, 66 Dafoe Road, Winnipeg, MB R3T 2N2, Canada; Zhongwei.Zou@umanitoba.ca

**Keywords:** *Camellia sinensis*, *NRAMP*, Pb treatment, gene expression

## Abstract

The natural resistant-associated macrophage protein (NRAMP) is a kind of integral membrane transporter which could function on a wide range of divalent metal ions in plants. Little is known about the NRAMP family in *Camellia sinensis*. In this study, 11 *NRAMP* genes were identified from the tea plant genome. Phylogenetic analysis showed that the 11 CsNRAMP proteins were split into two groups. The proteins of group 1 contained the conserved motif 6 (GQSSTxTG), while most proteins in group 2 (excepting CsNRAMP7 and CsNRAMP10) contained the conserved residues of motif 6 and motif 2 (GQFIMxGFLxLxxKKW). The number of amino acids in coding regions of 11 *CsNRAMP* genes ranged from 279–1373, and they contained 3–12 transmembrane domains. Quantitative RT-PCR analysis showed that G1 genes, *CsNRAMP3*, *CsNRAMP4*, and *CsNRAMP5*, were extraordinarily expressed in roots, while G2 genes showed higher expression levels in the stems and leaves. The expression levels of *CsNRAMPs* in roots and leaves were detected to assess their responses to Pb treatment. The results indicated that *CsNRAMPs* were differentially regulated, and they might play a role in Pb transportation of tea plant. Subcellular localization assay demonstrated that CsNRAMP2 and CsNRAMP5 fused proteins were localized in the plasma membrane. Overall, this systematic analysis of the *CsNRAMP* family could provide primary information for further studies on the functional roles of *CsNRAMPs* in divalent metal transportation in tea plants.

## 1. Introduction

As one of the most popular nonalcoholic beverage crops, the tea plant has been widely cultivated throughout the world [1]. The tea plant can absorb various nutrients and essential elements from soil to maintain growth and development [2]. However, some toxic substances, such as Pb, are also accumulated, which could have adverse impacts on morphological, physiological, and biochemical properties in plants [3,4]. Lead, as one of the toxic heavy metals, could block the function of essential metals and induce the production of ROS [5]. It could destroy the electron transport chain and induce lipid peroxidation [6]. Furthermore, toxic effects may appear in other cell mechanisms, such as water balance, protein structure, and photosynthesis [7,8]. Additionally, 24.4% to 72% of the total lead content in the dried black tea could be released into the tea infusion [9]. In a 74-sample study, 17.57% of the samples had a higher concentration of Pb than the maximum limits in tea leaves [10]. Thus, Pb pollution could cause significant damage to the tea plant, and even be harmful for consumer health [11]. Due to the rapid development of modern industry, urban activities, and transportation, lead contamination in tea has become a major concern [12,13,14]. Therefore, it is important to study the mechanisms of lead absorption and transportation for Pb resistance and lessening in tea plants.

To minimize the toxicity of heavy metals, plants have developed a complex network that controls metal uptake, transport, and storage [5]. Under heavy metal stress, some plants could accumulate metal ions in the roots and restrict the root-to-shoot transport to alleviate the toxicity in the overground part. Plant cells show defense against these heavy metals by chelating the toxic ions through a metal-chelating protein, as well as transporting into vacuoles or out the cell through metal membrane transporters [15].

The natural resistant-associated macrophage protein (NRAMP) is a kind of integral membrane transporter which usually has 10–12 putative transmembrane domains (TMDs), with consensus of residues between TMD-8 and TMD-9 [16]. The NRAMP was firstly discovered in mice [17] and has been identified as a divalent metal transporter in bacteria, fungi, insects, plants, and mammals [18,19]. The NRAMP protein family in *Arabidopsis* was classified into two subfamilies based on phylogenetic analysis. AtNRAMP1 and AtNRAMP6 form group I, while other AtNRAMP proteins belong to group II [20].

The NRAMP protein in plants is involved in the transport of multiple divalent cations, such as Fe^2+^, Mn^2+^, Cu^2+^, Pb^2+^, and Cd^2+^ [21,22]. The roles of NRAMP proteins in plants have been analyzed in *Arabidopsis thaliana* [20], *Oryza sativa* [23], *Populus alba* [24], *Phaseolus vulgaris* [25], *Theobroma cacao* [26], and *Brassica napus* [27]. In *Arabidopsis*, AtNRAMP6 is an intracellular cadmium transporter [28], while AtNRAMP1 locates in the root plasma membrane and acts as a transporter for Mn. Additionally, AtNRAMP3 and AtNRAMP4 play a role in the transportation of Fe and Mn [29]. In rice, OsNRAMP3 and OsNRAMP5 have been identified as a transporter for Mn and Fe, respectively. OsNRAMP4 has been identified as the trivalent Al ion transporter [16]. Many papers have presented studies on the role of NRAMP proteins in Cd transport [16,23]. Overexpression of TtNRAMP1 could enhance the transport of Cd [30]. However, the role of the NRAMP protein family and the transport mechanisms for Pb in tea plants remain unknown.

In this study, we identified and characterized 11 *NRAMP* genes in the CSS reference genome database, and then the expression levels of 11 *CsNRAMP* genes were investigated in different tissues and under Fe, Mn, and Pb treatment. Also, we analyzed the subcellular location of CsNRAMP2 and CsNRAMP5. The characterization and expression analysis of the *CsNRAMP* family in tea plants could provide a theoretical basis for further studies of the response of the *CsNRAMP* family on Pb treatment in tea plants.

## 2. Results

### 2.1. Identification and Characteristics of CsNRAMPs

Thirteen candidate CsNRAMP full-length sequences containing the NRAMP domain were identified in a Blast search of the tea plant genome. However, TEA002429.1 and TEA017931.1 were excluded because they are homologous with ethylene-insensitive protein 2 (EIN2) [20]. Thus, we identified 11 *CsNRAMP* genes in this study (Table 1).

The number of amino acids of 11 CsNRAMP proteins ranged from 279 (CsNRAMP7) to 1373 (CsNRAMP11), while the molecular weight varied from 30,575.14–150,318.02 Da. The theoretical pIs of CsNRAMP proteins were various from 4.94–9.18. The exon numbers of CsNRAMPs ranged from 3–17. And the transmembrane domains of CsNRAMPs ranged from 3–12 (Table 1).

### 2.2. Phylogenetic Analysis and Duplication Analysis of CsNRAMPs

**Figure 1:**
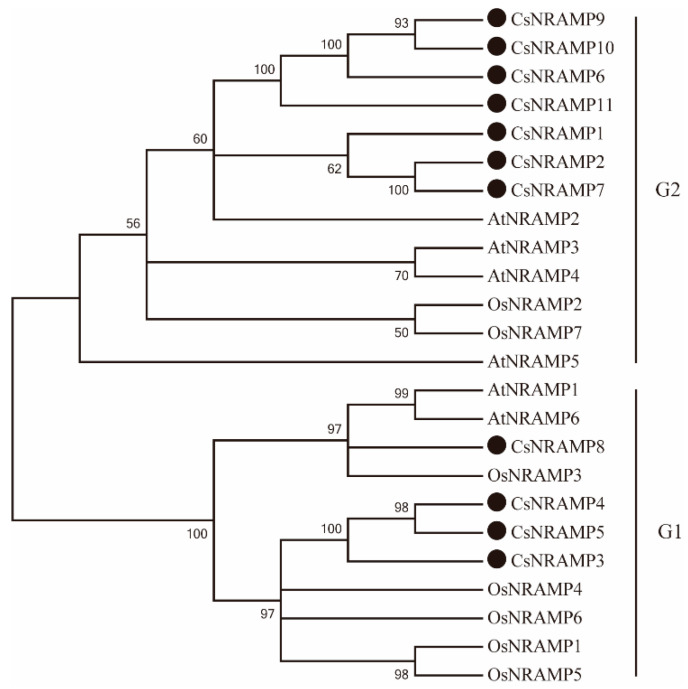
Unrooted phylogenetic tree analysis of NRAMP proteins from *Camellia sinensis* (Cs), *Arabidopsis thaliana* (At), and *Oryza sativa* (Os). Eleven sequences from *Camellia sinensis* are highlighted with a black circle. The two groups are indicated in the right panel.

To explore the phylogenetic association among NRAMP homologs in plant genomes, the sequences of 24 proteins from *Arabidopsis thaliana*, *Oryza sativa*, and *Camellia sinensis* were used to align a phylogenetic tree analysis. According to AtNRAMP proteins, the 11 CsNRAMP proteins were divided into two groups (Figure 1). CsNRAMP3, CsNRAMP4, CsNRAMP5, and CsNRAMP8 belonged to group 1, while other CsNRAMPs were included in group 2. Additionally, it was predicted that the CsNRAMPs of G1 (group 1) would locate on the plasma membrane, and the CsNRAMPs of G2 (group 2) on the membrane-bound vacuolar. The exon numbers of G1 CsNRAMPs varied from 12–13, and the AtNRAMP1 and AtNRAMP6 from the same group displayed 11 and 13 exons [25]. In another group, most members contained no more than five exons, which was similar to those for which the exon numbers were restricted to four [25] (Table 1).

**Figure 2:**
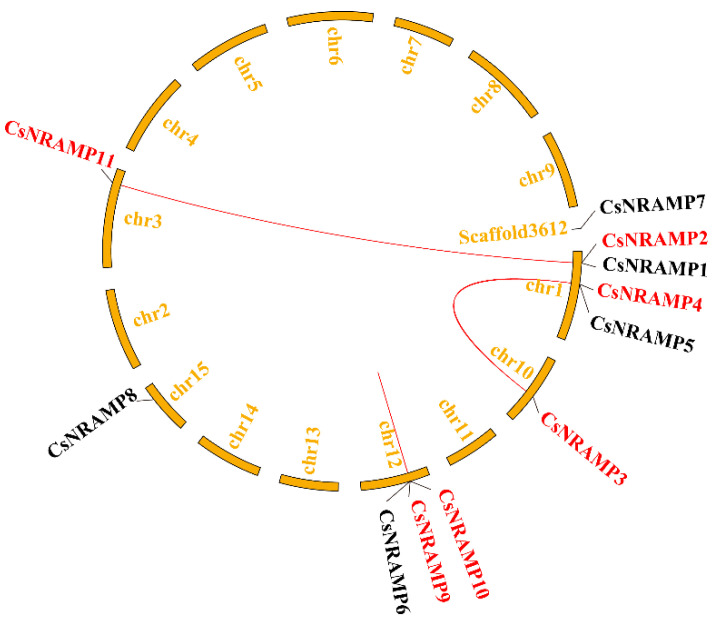
Duplication analysis of *CsNRAMP* genes. The red lines mean duplicated *CsNRAMP* gene pairs.

To explore the evolutionary patterns of the *NRAMP* gene family in the tea plant genome, gene duplication events were surveyed (Figure 2). Ten *NRAMP* genes were distributed unevenly in the 15 chromosomes in the tea plant genome. *CsNRAMP7* was assembled to the scaffold. An analysis of tea plant *NRAMP* family genes revealed that three paralogous gene pairs (*CsNRAMP2&CsNRAMP11/CsNRAMP3&CsNRAMP4/CsNRAMP9&CsNRAMP10*) existed in *CsNRAMP* family genes. (The ‘&’ means the connector between duplicated gene pairs.)

### 2.3. Gene Structure and Conserved Motifs of CsNRAMP Proteins

**Figure 3:**
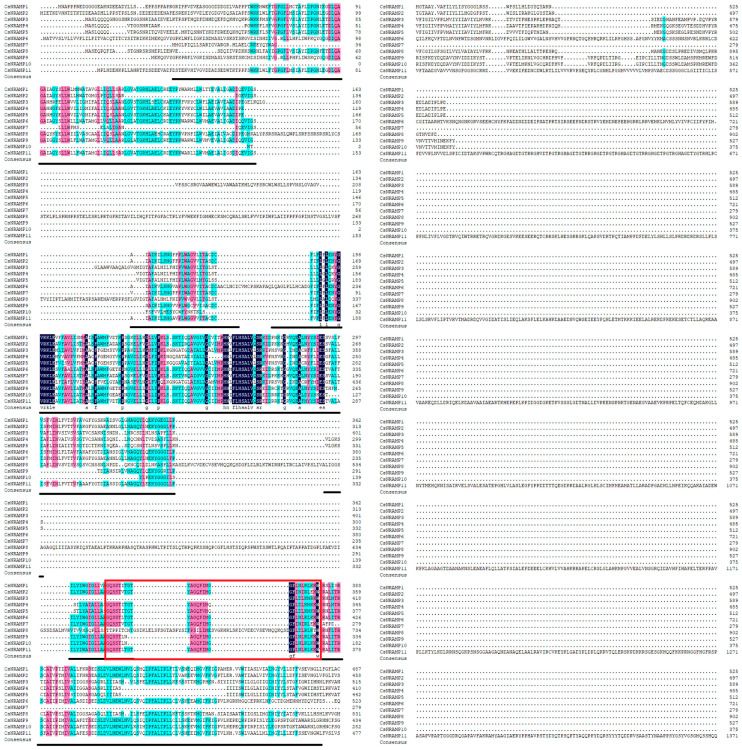
Multiple alignments of CsNRAMP proteins. The residues of NRAMP domain were underlined. And red rectangular frames mean the conserved residues.

Previous studies on Arabidopsis and rice indicated that the NRAMP protein family usually contained 10–12 TMDs. Additionally, the most conserved amino acid residues, GQSSTITGGTYAGQXXMXGFLX, were located between the eighth and ninth transmembrane domains [31]. Most of the CsNRAMPs contained the conserved amino acid residues GQSSTxTGTYAGQFI MxGFLxLxxKKW, while CsNRAMP3 only had the latter part of residues, while CsNRAMP8 contained some other residues (Figure 3). Some NRAMP proteins contained broken NRAMP domains (Supplementary Materials, Figure S1).

**Figure 4:**
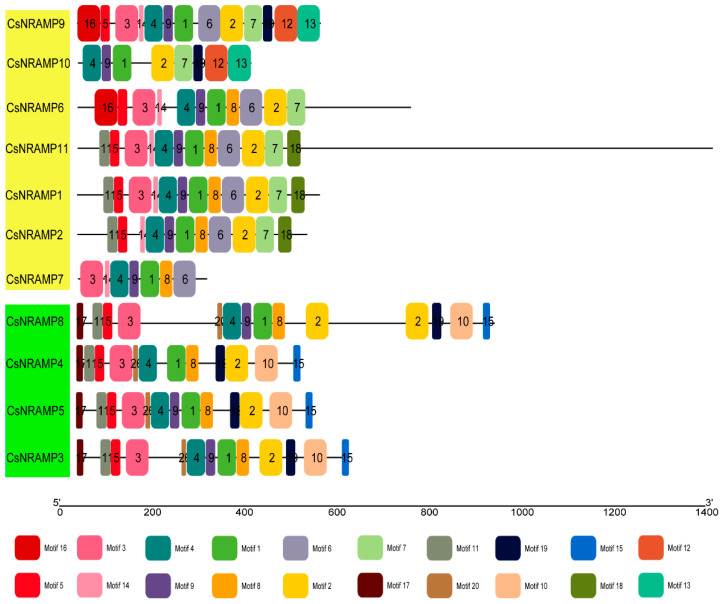
Motif analysis of CsNRAMP proteins. Boxes in different colors are used to distinguish different motif. CsNRAMP proteins were divided into two groups with yellow and green colors based on phylogenetic tree analysis.

**Figure 5:**
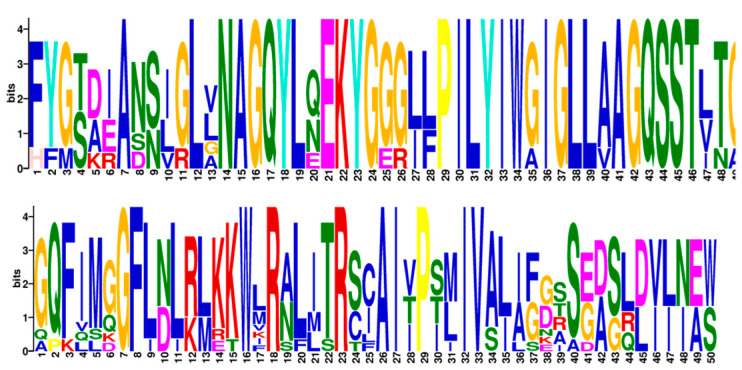
Sequence logos of conserved amino acid residues sequences in *Camellia sinensis*. The above one indicates motif 6, the bottom one means motif 2. MEME motifs are displayed by stacks of letters at each site. The x-axis represents the width of the motif and the y-axis represents the bits of each letter.

There were 20 motifs identified in CsNRAMP proteins. The sequences of 20 motifs are shown in Supplementary materials, Table S1. All CsNRAMP proteins contained motif 1 and 4, while most of the CsNRAMP proteins in group 2 had motif 2, 3, 5, 6, 7, 8, and 14; and all of CsNRAMP proteins in group 1 had motif 2, 3, 5, 8, 10, 11, 15, 17, 19 and 20 (Figure 4). Only CsNRAMP9 and CsNRAMP10 proteins contained motif 12 and motif 13; motif 16 only existed in CsNRAMP6 and CsNRAMP9 proteins and motif 18 only existed in CsNRAMP1, CsNRAMP2 and CsNRAMP11 proteins. The conserved amino acid residues (Figure 3) consisted of motif 2 (GQFIMxGFLxLxxKKW) and motif 6 (GQSSTxTG) (Figure 5). All members of group 1 lacked the conserved motif 6 (GQSSTxTG).

### 2.4. Expression Analysis of CsNRAMP Genes in Different Tissues

**Figure 6:**
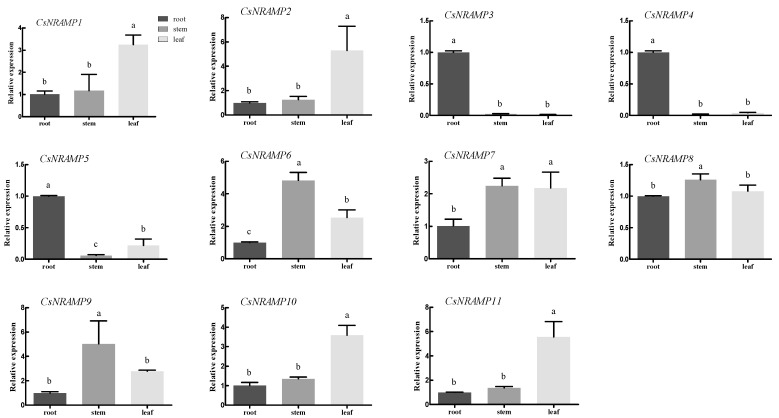
Expression of 11 *CsNRAMP* genes of *Camellia sinensis* in different tissues (root, stem, and leaf) of the tea plant. The expression level of tea actin was used as the internal control to standardize the RNA samples for each reaction. Error bars represent the mean values of three replicates ± standard deviation (SD). Different lowercase letters indicate significant differences at *p* < 0.05.

We detected the expression of 11 *CsNRAMP* genes in different tissues of the tea plant. The results indicate that different genes exhibit different expression patterns (Figure 6). Three genes, *CsNRAMP3*, *CsNRAMP4*, and *CsNRAMP5*, showed extremely high specificity in the root, while four, *CsNRAMP1*, *CsNRAMP2*, *CsNRAMP10*, and *CsNRAMP11*, were highly expressed in the leaf, and *CsNRAMP6* and *CsNRAMP9* were highly expressed in the stem. Also, two genes, *CsNRAMP7* and *CsNRAMP8*, exhibited high expression in both shoot and leaf. The genes in group 1, including *CsNRAMP3*, *CsNRAMP4*, and *CsNRAMP5*, showed similar expression patterns and reached higher expression levels in the root. Conversely, the genes belonging to group 2 were highly expressed in other tissues, such as stem and leaf. The CsNRAMP proteins might play different roles in the transportation of metals.

### 2.5. Expression Analysis of CsNRAMP Genes under Lead Stress

To investigate potential responses of *CsNRAMPs* to Pb treatment, expression patterns were detected by qRT-PCR in tea plants exposed to Pb. We noticed that *CsNRAMP1,* which prefers to express in leaves, showed an extremely low expression level in roots, while *CsNRAMP3 CsNRAMP4* and *CsNRAMP5* exhibited extremely low expression in leaves under Pb treatment.

**Figure 7:**
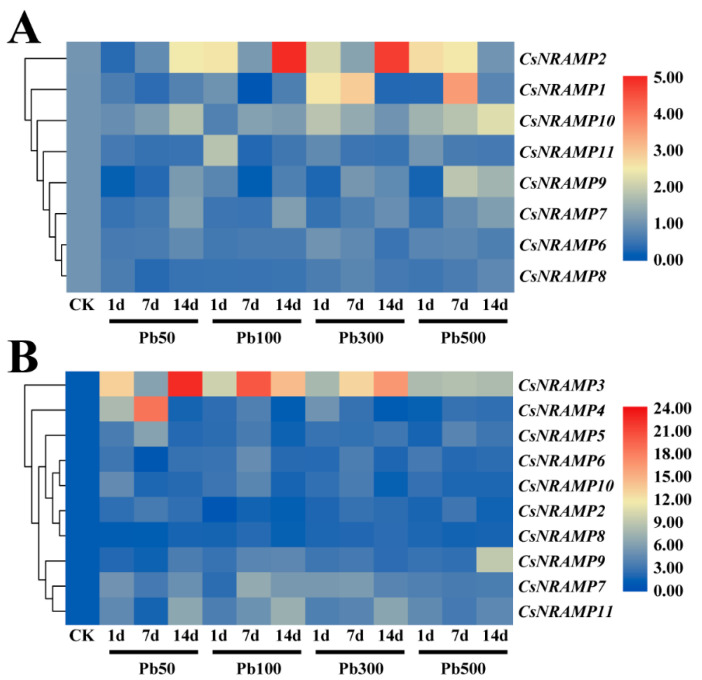
Expression of 11 *CsNRAMP* genes of *Camellia sinensis* under Pb treatment. (**A**) Expression heatmap of 8 *CsNRAMP* genes under different concentrations of Pb treatment in leaves. (**B**) Expression heatmap of 10 *CsNRAMP* genes under different concentrations of Pb treatment in roots. The expression level of tea actin was used as the internal control to standardize the RNA samples for each reaction and the expression at 0 day was set as CK. The underline with marks of Pb50, Pb100, Pb300, and Pb500 indicated that the concentration of Pb treatment was 50 mg/L, 100 mg/L, 300 mg/L, and 500 mg/L, respectively. And the sampling time after each treatment was 1 day, 7 days, and 14 days. Relative expression levels were shown in color as the scale.

In leaves, the expression of *CsNRAMP2* was downregulated earlier and then upregulated at 50 mg/L Pb treatment, and showed a double-peak pattern at 100 mg/L Pb treatment. Additionally, *CsNRAMP2* was significantly induced at 500 mg/L Pb treatment for 1 and 7 days. The expression of *CsNRAMP1* was downregulated under low Pb concentrations (≤100 mg/L), but increased at first and then decreased at high concentration. *CsNRAMP10* showed a pattern that decreased and then increased at low Pb concentration, while it could be induced by high concentration. *CsNRAMP11* expression, except at 100 mg/L for 1 day, was downregulated by Pb treatment. The expression of *CsNRAMP7* and *CsNRAMP9* exhibited a tendency that was downregulated and then increased. The expression of *CsNRAMP6* and *CsNRAMP8* was decreased (Figure 7A and Supplementary Materials, Figure S2).

In roots, *CsNRAMP3* was significantly expressed in response to lead stress, and showed a double-peak pattern. At 50 and 100 mg/L Pb treatment, the expression of *CsNRAMP4* and *CsNRAMP5* was upregulated and showed the highest transcriptional levels at 7 days, before decreasing. However, *CsNRAMP4* and *CsNRAMP5* were induced and stably expressed at 500 mg/L and 300 mg/L Pb treatment. The *CsNRAMP6* and *CsNRAMP10* showed an initial increase, but subsequently decreased under Pb treatments. *CsNRAMP2* and *CsNRAMP8* showed a slight increase after treatment. The expression of *CsNRAMP7*, *CsNRAMP9*, and *CsNRAMP11* increased, and *CsNRAMP9* was highly expressed at 500 mg/L for 14 days. (Figure 7B and Supplementary Materials, Figure S3).

### 2.6. Cloning of CsNRAMP2 and CsNRAMP5 and Subcellular Localization

**Figure 8:**
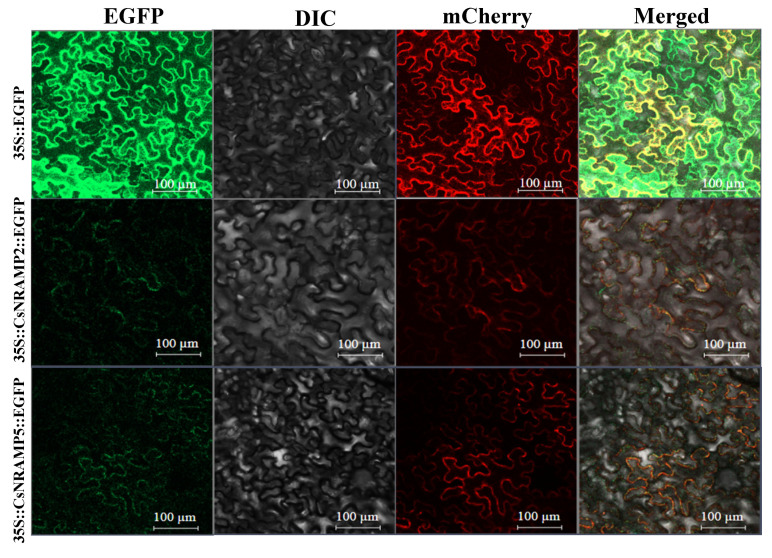
Subcellular location of the fusion protein 35S::CsNRAMP2::EGFP and 35S::CsNRAMP2::EGFP in tobacco epidermis cells (through transient infiltration). The vector 35S::EGFP was used as the control, and the AtPIP2A-mCherry was used as a plasma membrane (PM) marker. Bar = 100 μm.

To understand the subcellular location of the CsNRAMP2 and CsNRAMP5 proteins, full-length *CsNRAMP2* and *CsNRAMP5* without ending codes were cloned and inserted into EGFP-fusion expression vector. The recombinant plasmid with a plasma membrane (PM) marker *AtPIP2A-mCherry* was cotransformed through transient infiltration to tobacco epidermis cells. The results indicated that both EGFP-CsNRAMP2 and EGFP-CsNRAMP5 fusion proteins were located in the plasma membrane (Figure 8). The subcellular localization of EGFP-CsNRAMP5 was coincident with the result predicted on the SoftBerry ProtComp website. However, the subcellular localization of CsNRAMP2 was the plasma membrane, which was different from the prediction of the SoftBerry ProtComp website.

## 3. Discussion

Based on the conserved domain of AtNRAMPs (PF01566), we identified 11 *NRAMP* genes from the Tea Plant Genome Database. Since the *NRAMP* genes were divided into two groups in *Arabidopsis,* we also split the *CsNRAMPs* into two groups [20]: *CsNRAMP3, CsNRAMP4, CsNRAMP5*, and *CsNRAMP8* in group 1, and other *CsNRAMPs* in group 2.

The NRAMP proteins have been reported to contain 10–12 transmembrane domains and consist of around 500 amino acid residues in different species. For example, OsNRAMP proteins contained 518–550 amino acid residues and 10–12 transmembrane regions in rice [16]. Similarly, it was verified that there are 12 transmembrane domains in PvNRAMPs, and that the length of amino acid residues ranged from 507–554 [25]. However, in this study, we learned that CsNRAMP proteins contain 3–12 transmembrane regions and consist of 279–1373 amino acid residues. This may have been due to a broken NRAMP domain (Supplementary materials, Figure S1) or variation among species. This result was similar to those of several members of BnNRAMPs, which only carried 100–200 amino acid residues [29]. NRAMP proteins have been reported to carry consensus residues between TMD8 and TMD9, e.g., the AtNRAMP proteins in *Arabidopsis* contained GQSSTITGTY AGQXXMXGFLX, while PvNRAMP proteins in *Phaseolus vulgaris* carried GQSSTITGTYAGQFIMGGFLN [25,28]. According to our results, the CsNRAMP proteins carried similar consensus residues, i.e., GQSSTxTGTYAGQFIMxGFLxLxxKKW, which consisted of motif 2 (GQFIMxGFLxLxxKKW) and 6 (GQSSTxTG). For motif analysis, motifs 6 and 14 are only present in G2 CsNRAMP proteins, while motifs 10, 15, 17, and 20 are only present in the G1 CsNRAMP proteins; this may be related to the differences between groups. We also analyzed motifs of the tea plant, Arabidopsis and rice, finding that motifs 17 and 18 discriminated groups 1 and 2 (Supplementary Materials, Figure S4). This was similar to the results of NRAMPs of cacao, Arabidopsis, and rice, which were split into three clusters with a comparison of conserved motifs [26].

In this study, we learned that G1 genes, *CsNRAMP3*, *CsNRAMP4*, and *CsNRAMP5*, were extraordinarily expressed in roots, while G2 genes showed higher expression levels in the stems and leaves. The *NRAMP* family genes of plants have been proven to function on a wide range of divalent metal ions, including absorption, transportation, and homeostasis [32]. For example, AtNRAMP6 functions in the lateral root and young leaves of *Arabidopsis* [33]. AtNRAMP1 plays a pivotal role in the Fe transportation in roots [34]. *SaNRAMP1* can be strongly expressed in the young shoots and transportation of Cd, Mn, and Zn [35]. To sum up, the members of G1 *CsNRAMPs* were mainly expressed in the root, combing with their plasma membrane localization (Table 1), indicated that G1 *CsNRAMPs* may participate in the absorption process of metal ions. AtNRAMP3 and AtNRAMP4 are located on vacuolar membranes and function in vacuolar Fe mobilization [36,37], while it was predicted that the G2 members would be located on the membrane bound vacuolar, which might be related to the transport and distribution of metal in the tea plant.

In plants, NRAMPs were found to participate in multiple divalent metal transportation [22]. The expression of BnNRAMP2;1 and BnNRAMP4;2 could be increased after exposure to Cd [38]. Nrat1 characterized as Al transporters in rice could be highly expressed in response to Al stress [39]. *LeNRAMP1* could be significantly induced by iron deficiency [40]. AtNRAMP3 and AtNRAMP4 proteins have been proved to function in the transportation of Fe and Mn [36,37]. OsNRAMP6 and OsNRAMP5 have been shown to be involved in the uptake of Fe and Mn [16]. According to our results, the members of *CsNRAMPs* could be differently upregulated or downregulated under Pb treatment. The expression levels of most *CsNRAMPs* in leaves were lower than in roots. This may have been due to the fact that plants restrict most of the heavy metals to the roots to alleviate toxicity on shoots and leaves [15]. In leaves, the expression levels of *CsNRAMP1*, *CsNRAMP2*, *CsNRAMP9*, and *CsNRAMP10* were upregulated, and *CsNRAMP2* showed extremely sensitive response to Pb treatment. In roots, the transcription levels of *CsNRAMP3*, *CsNRAMP4*, *CsNRAMP5*, *CsNRAMP7*, and *CsNRAMP9* were accumulated after exposure to Pb. In particular, the expression of *CsNRAMP3* was sharply increased under Pb treatment. This indicates that these genes may play a role in Pb transportation. Interestingly, some *CsNRAMPs* showed a double-peak pattern after Pb treatment, which may be have been due to a complicated interaction mechanism in the tea plants. The MTs also showed a similar expression pattern under Cd stress [41].

Regarding G2 members, the NRAMP domain of CsNRAMP3 protein was broken (Supplementary Materials, Figure S1). CsNRAMP7, CsNRAMP9, and CsNRAMP10 only contained six, six, and three transmembrane regions respectively, in contrast to 10–12 TMDs. Furthermore, CsNRAMP1 and CsNRAMP2 were close on the phylogenetic tree, and the expression level of *CsNRAMP2* increased more than *CsNRAMP1* under Pb treatment in leaves. Regarding G1 members, both expression levels *CsNRAMP4* and *CsNRAMP5* were increased by Pb treatment in roots; however, the expression of *CsNRAMP5* under 100 mg/L Pb treatment for 14 days was increased around 12 fold in leaves (Supplementary Materials, Figure S2). Overall, *CsNRAMP2* from G2 and *CsNRAMP5* from G1 were chosen for further studies, since *NRAMP* genes function in metal ion transportation, especially Fe [42]. In this study, the responses of *CsNRAMP2* and *CsNRAMP5* to Fe and Mn treatments were studied. The results showed that Fe treatment (400 μM EDTA-Fe) increased the expression of *CsNRAMP2* and decreased the expression of *CsNRAMP5*, while Mn treatment up-regulated the expression of *CsNRAMP5* in roots (Supplementary Materials, Figure S5). As shown in Figure 1, the CsNRAMP2 protein clustered together into a small phylogenetic branch with AtNRAMP2, AtNRAMP3, and AtNRAMP4, which are known to be implicated in the transportation of Fe and Mn [36,37]. Thus, *CsNRAMP2* may play a role in transporting Fe and Pb. Meanwhile, CsNRAMP5 was close to OsNRAMP6 and OsNRAMP5, which have been shown to be involved in the uptake of Fe and Mn [16]. *CsNRAMP5* may participate in the transportation of Mn and Pb. All of these speculations need further study.

Proteins of the NRAMP family located on different organelles show various functions. For example, AtNRAMP6 has been shown to be located in the Golgi/trans-Golgi network and play an important role in intracellular Fe homeostasis [33]. Additionally, AtNRAMP3 and AtNRAMP4 are located on vacuolar membranes and contribute to Fe mobilization [37]. OsNRAMP1 on the plasma membrane of endodermis and pericyle cells may assist in the loading of arsenic from roots to shoots mobilization [32]. In this study, we analyzed the subcellular locations of the CsNRAMP2 and CsNRAMP5 proteins. The results indicated that both CsNRAMP2 and CsNRAMP5 fusion proteins are located in the plasma membrane. CsNRAMP2 and CsNRAMP5 fusion proteins may function in the transmembrane transport of metal ions.

In summary, we first identified 11 *NRAMP* genes in a genome-wide survey of the tea plant. The expression profiles of *CsNRAMP* genes varied in tea plant tissues, implying that *CsNRAMPs* perform functions in specific tissues. Based on the expression analysis under Pb treatment, we speculated that certain *CsNRAMP* genes might contribute to Pb transportation. Furthermore, the subcellular localization analysis in tobacco epidermis cells confirmed the plasma membrane localization of CsNRAMP2 and CsNRAMP5 proteins. These results provide basic information for understanding the functions of the NRAMP family in tea plants, and suggest potential future study directions regarding transport signaling pathways.

## 4. Materials and Methods

### 4.1. Identification of CsNRAMP Family Genes in the Tea Plant

The sequences of *NRAMP* genes of the tea plant were obtained derived from the Tea plant Genome Database (http://tpia.teaplant.org/download.html (accessed on 1 October 2019)). The NRAMP domain (PF01566) of six AtNRAMP proteins was used to search the local tea protein database for target sequences by using Bioedit version 7.0.9 software. Pfam Database (http://pfam.xfam.org/ (accessed on 30 October 2019)) and SMART (http://smart.embl-heidelberg.de/ (accessed on 30 October 2019)) were used to determine predicted protein as a member of the transporter gene family.

### 4.2. Characterization of CsNRAMP Proteins

Six *Arabidopsis* NRAMP protein sequences and seven rice NRAMP protein sequences were downloaded from the Arabidopsis Information Resource (TAIR) (https://www.arabidopsis.org/ (accessed on 23 December 2020)) and The Rice Annotation Project Database (https://rapdb.dna.affrc.go.jp/download/irgsp1.html (accessed on 23 December 2020)). MEGA version 7.0 was used to construct a bootstrap neighbor-joining(NJ) phylogenetic tree for NRAMP protein of *Arabidopsis*, rice, and tea with MUSCLE alignment and 1000 bootstrap replicates [43]. The duplication events were analyzed using the DupGen_finder [44], and then visualized using TBtools v.1.0692 [45]. The physicochemical properties of the CsNRAMP proteins were analyzed using ExPASy-ProtParam (https://web.expasy.org/protparam/ (accessed on 22 July 2020)). The exon organization was determined using by TBtools v.1.0692 [45]. Prediction of subcellular localization and transmembrane helices of the proteins encoded by *CsNRAMP* genes were displayed by SoftBerry ProtComp (http://linux1.softberry.com/berry.phtml?topic=protcomppl&group=programs&subgroup=proloc (accessed on 22 July 2020)) and TMHM M Server v.2.0 (http://www.cbs.dtu.dk/services/TMHMM-2.0/ (accessed on 22 July 2020)). The conserved motifs and domains of CsNRAMP proteins were obtained using the MEME (E < 1e^−10^) online tool (http://meme-suite.org/tools/meme (accessed on 5 January 2021)) and Pfam (http://pfam.xfam.org/ (accessed on 5 January 2021)) respectively, and then visualized by visualizing the domain pattern of TBtools v.1.0692 [45]. Finally, multiple sequence alignments of the CsNRAMP proteins were performed using DNAMAN version 7.0.

### 4.3. Plant Materials and Treatments

One-year-old seedlings of tea plants “zhongcha108” were grown in a growth chamber at the Tea Science Research Institute of Nanjing Agricultural University (Nanjing, China). These seedlings were cultivated in a nutrient solution [46], and were precultured in 1/4 nutrient solution and then 1/2 nutrient solution for acclimatization. Then, seedlings were cultured in a total nutrient solution for about two weeks. After that, the leaves, stems, and roots were harvested for expression analysis of *CsNRAMP* genes of the tea plant.

Regarding lead treatment, the seedlings were cultivated in a nutrient solution containing 50, 100, 300, 500 mg/L Pb^2+^ (Pb(NO_3_)_2_), and then the leaves and roots were collected at 1, 7, and 14 days after treatment. The CK leaves and roots were collected before lead treatment.

To investigate the possible function of *CsNRAMP2* and *CsNRAMP5* in response to divalent metal, tea plant seedlings were exposed to the treatments of iron and manganese for 7 days, respectively. For iron treatment, excess Fe (200 μM or 400 μM EDTA-Fe) was added for iron treatment. Additionally, 200 μM or 400 μM MnSO_4_·H_2_O was added for Mn treatment. The seedlings cultivated in full-strength nutrient solution were sampled as the control (CK, 6.27 μM Fe-Na-EDTA and 18.22 μM MnSO_4_·H_2_O).

All samples were frozen in liquid nitrogen, then stored at −80 ℃ for the following experiments. All experiments were repeated with three biological and technical replicates.

### 4.4. Expression Profile Analyses

Total RNA was extracted from the leaves and roots using an RNA quick isolation kit (Aidlab, Beijing, China), and reverse transcription was performed with TransScript^®^ One-Step gDNA Removal and cDNA Synthesis SuperMix (TransGen, Beijing, China). The expression of *CsNRAMPs* was analyzed by quantitative real-time PCR using the SYBR Premix Ex Taq II kit (Takara, Kusatsu, Japan). The primer pairs used for qRT-PCR were designed by Primer Premier 5.0 and β-actin was used as an internal control (Table S2). The qRT-PCR program was as follows: 95 ℃ for 30 s, 40 cycles at 95 ℃ for 5 s, 60 ℃ for 30 s [47]. All experiments were repeated with three biological and technical replicates. Relative gene expressions were calculated using the 2^−∆∆Ct^ method [48].

### 4.5. Subcellular Location Confirmation of CsNRAMP2 and CsNRAMP5

The full open-reading frame sequences of *CsNRAMP2* and *CsNRAMP5* gene without the stop codon were amplified and then inserted into EGFP-fusion expression vector pCAMBIA 2300 at XbaⅠ and SmaⅠ site using Trelief^TM^ SoSoo Cloning Kit Ver.2 (Tsingke, Beijing, China). The recombinant plasmid, *EGFP-CsNRAMP2* or *EGFP-CsNRAMP5*, and empty vector were transformed into *Agrobacterium tumefaciens* strain GV3101 cells. Furthermore, we cotransformed GV3101 containing recombinant plasmids or empty vectors with a plasma membrane (PM) marker *AtPIP2A-mCherry* through transient infiltration to tobacco epidermis cells [49]. Plants were incubated in the dark overnight and normal cycle for two days and then detected using An LSM800 Ultra high-resolution confocal microscopy imaging system (Zeiss Co., Oberkochen, Germany) [50].

### 4.6. Statistical Analysis

Excel 2010, SPSS 20.0, and GraphPad Prism 5 were used to analyze the experimental data. Duncan’s method was used for multiple comparisons of variance analysis, and *p* < 0.05 indicated a significant difference.

## Figures and Tables

**Table 1:** Bioinformatics analysis of *CsNRAMP* genes.

Name	Gene ID	Number of Amino Acids	Molecular Weight	Theoretical pI	Exon Number	Transmembrane Number	Subcellular Localization
CsNRAMP1	TEA000600.1	525	57,407.13	5.23	4	11	Membrane bound Vacuolar
CsNRAMP2	TEA000624.1	497	54,044.99	4.94	5	9	Membrane bound Vacuolar
CsNRAMP3	TEA009385.1	589	64,295.88	8.75	13	10	Plasma membrane
CsNRAMP4	TEA002435.1	485	52,482.32	8.81	12	11	Plasma membrane
CsNRAMP5	TEA017264.1	512	56,070.19	8.05	12	11	Plasma membrane
CsNRAMP6	TEA012361.1	721	80,236.18	8.55	10	11	Membrane bound Vacuolar
CsNRAMP7	TEA012584.1	279	30,575.14	9.18	3	6	Membrane bound Vacuolar
CsNRAMP8	TEA022476.1	902	98,625.55	9.01	17	14	Plasma membrane
CsNRAMP9	TEA025235.1	527	58,851.23	8.17	5	6	Membrane bound Vacuolar
CsNRAMP10	TEA032256.1	375	42,411.81	8.36	5	3	Membrane bound Vacuolar
CsNRAMP11	TEA011223.1	1373	150,318.02	5.81	10	12	Membrane bound Vacuolar *

*: asterisks correspond to the predicted result of the first 1200 amino acids of CsNRAMP11.

## Data Availability

Not applicable.

## Supplementary Materials

The following are available online at https://www.mdpi.com/article/10.3390/plants10061055/s1, Figure S1: Domain analysis of CsNRAMP proteins. Figure S2: Expression of *CsNRAMP2*, *-4* and *-5* of *Camellia sinensis* in leaves. Figure S3: Expression of *CsNRAMP1* of *Camellia sinensis* in roots. Figure S4 Motif analysis of NRAMP proteins from *Camellia sinensis* (Cs), *Arabidopsis thaliana* (At), and *Oryza sativa* (Os). Figure S5 Expression of *CsNRAMP2* and *CsNRAMP5* of *Camellia sinensis* (leaf and root) in response to Fe and Mn treatments. Table S1: Motif consensus. Table S2: Primers for expression analysis.
