# Supplementary material for: Genome-Wide Identification and Expression Analysis of the NRAMP Family Genes in Tea Plant (Camellia sinensis)
Source: Plants (Basel). 2021 May 25;10(6):1055. doi: 10.3390/plants10061055 (PMC8228228; doi:10.3390/plants10061055)
Supplement: Supplementary file 1 [file plants-10-01055-s001.zip › Figure Supplementary-20210429.pdf]

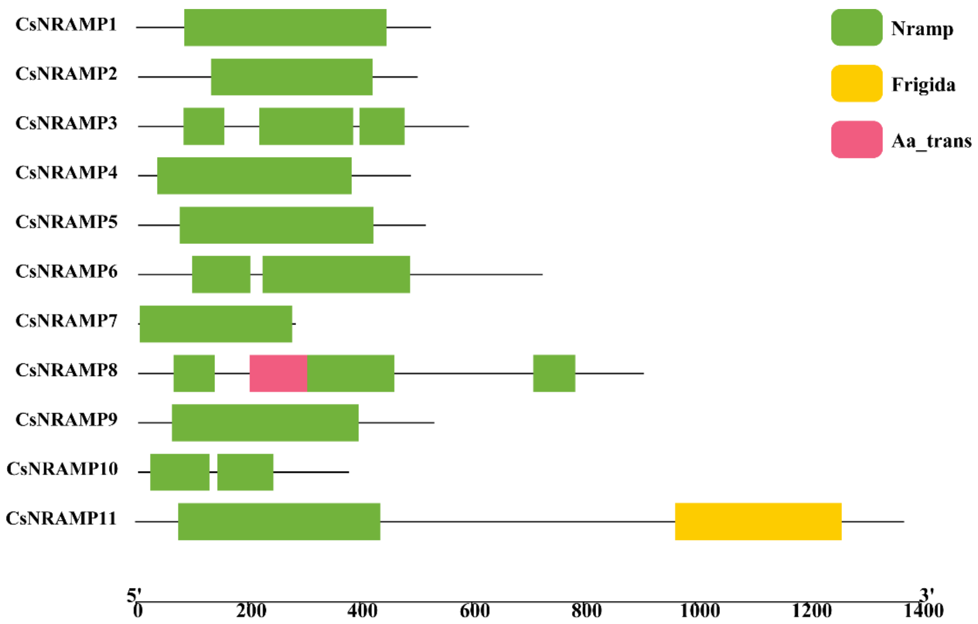

Fig. S1 Domain analysis of CsNRAMP proteins. Boxes in different color used to distinguish different domains.

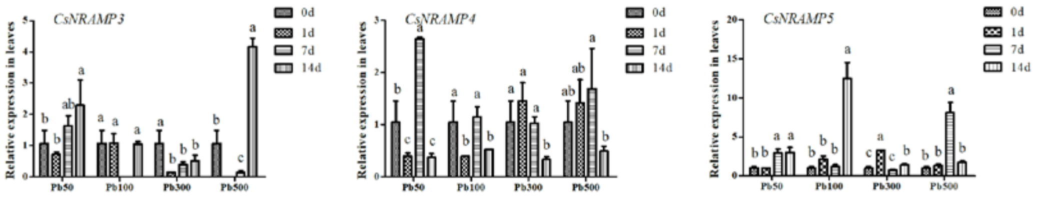

Fig. S2 Expression of *CsNRAMP2*, *-4* and *-5* of *Camellia sinensis* in leaves. The expression level of tea actin was used as the internal control to standardize the RNA samples for each reaction and the expression at 0 day was set as CK. The marks Pb50, Pb100, Pb300, and Pb500 indicated that the concentration of Pb treatment was 50 mg/L, 100 mg/L, 300 mg/L, and 500 mg/L, respectively. And the sampling time after each treatment were 1 day, 7 days, and 14 days. Error bars represent the mean values of three replicates ± standard deviation (SD). Different lowercase letters indicate significant differences at $P < 0.05$.

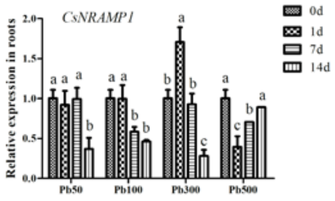

Fig. S3 Expression of *CsNRAMP1* of *Camellia sinensis* in roots. The expression level of tea actin was used as the internal control to standardize the RNA samples for each reaction and the expression at 0 day was set as CK. The marks Pb50, Pb100, Pb300, and Pb500 indicated that the concentration of Pb treatment was 50 mg/L, 100 mg/L, 300 mg/L, and 500 mg/L, respectively. And the sampling time after each treatment were 1 day, 7 days, and 14 days. Error bars represent the mean values of three replicates ± standard deviation (SD). Different lowercase letters indicate significant differences at $P < 0.05$.

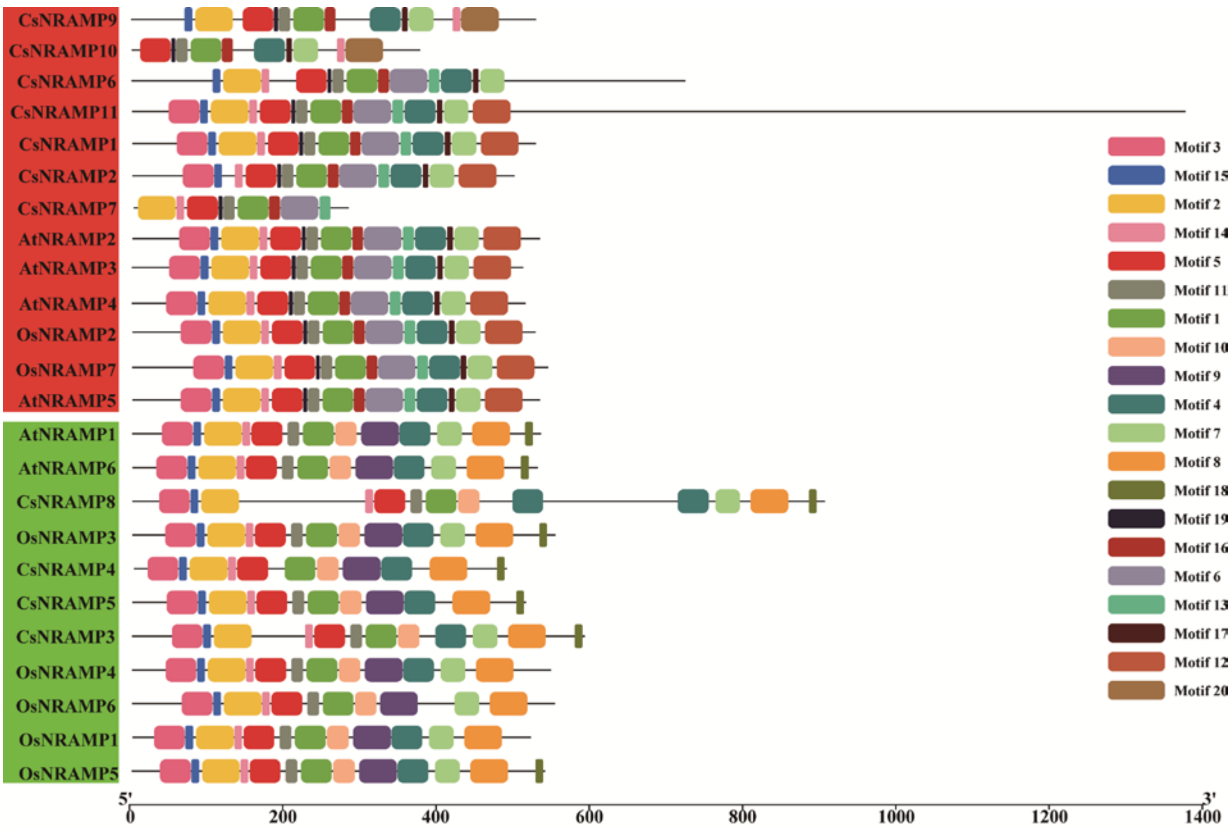

Fig. S4 Motif analysis of NRAMP proteins from *Camellia sinensis* (Cs), *Arabidopsis thaliana* (At), and *Oryza sativa* (Os). Boxes in different colors are used to distinguish different motif. The NRAMP proteins were divided into two groups with red and green colors based on phylogenetic tree analysis.

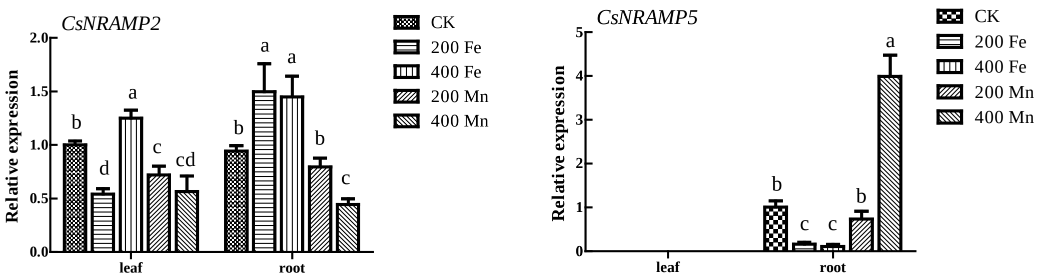

Fig. S5 Expression of *CsNRAMP2* and *CsNRAMP5* of *Camellia sinensis* (leaf and root) in response to Fe and Mn treatments. The tea plant seedlings were treated with Fe (200 Fe: 200 μM EDTA-Fe; 400 Fe: 400 μM EDTA-Fe), and Mn (200 Mn: 200 μM $MnSO_4 \cdot H_2O$; 400 Mn: 400 μM $MnSO_4 \cdot H_2O$) for 7 days. The tea plant seedlings cultured in full-strength nutrient solutions were used as control (CK, 6.27 μM Fe-Na-EDTA and 18.22 μM $MnSO_4 \cdot H_2O$). Error bars represent the mean values of three replicates ± standard deviation (SD). Different lowercase letters indicate significant differences at $P < 0.05$.
